# Special Issue “Gamma Delta T Cells in Immune Response against Viruses”

**DOI:** 10.3390/v14040736

**Published:** 2022-03-31

**Authors:** Eric Champagne

**Affiliations:** Infinity, Université Toulouse, Centre National de la Recherche Scientifique (CNRS), Institut National de la Santé et de la Recherche Médicale (INSERM), Université Paul Sabatier, CEDEX 03, 31024 Toulouse, France; eric.champagne@inserm.fr; Tel.: +33-562-748-369

γδ T cells are members of ‘unconventional’ T cells that combine the properties of adaptive T lymphocytes and innate cells such as NK cells. Although they represent a quantitatively minor population of the circulating lymphocytes, they contribute to all immune processes such as anti-cancer immunity, autoimmune diseases and defence against infection by all kinds of pathogens. The fundamental aspects of their antigen recognition are not yet fully understood, and they are often considered to be an archaic immune defence system. Nevertheless, over the last decade, major molecular targets involved in their function have been discovered, and it is increasingly clear that they have been conserved across multiple animal species, with species-specific diversification probably reflecting environmental adaptation. This Special Issue brings together four papers contributing to a better definition of their involvement in human viral infections. Among viruses, a distinction must be made between those that lead to persistent infections, such as those of the herpes virus family (notably CMV and EBV), and those that are responsible for acute pathologies. It is found that γδ T cell alteration/mobilisation is almost constantly observed, although with variations.

Two original studies [1,2] deal with acute dengue virus (DV) infection and SARS-CoV-2, respectively. Although using different approaches, either FACS [1] or transcriptomics [2], these studies reveal that both conditions are associated with peripheral γδ T cell depletion. During DV infection, however, the remaining γδ T cells show markers of activation and exhaustion. Whether peripheral depletion is due to deletion or recruitment to tissues is unknown. In SARS-CoV-2 infections [2], single-cell transcriptome analysis was used to correlate markers of activation, differentiation and tissue residence with the T cell receptor characteristics of γδ T cell subsets. In addition to demonstrating the depletion of γδ T cells in the blood, this analysis revealed the presence of tissue recirculation markers on the remaining cells. Importantly, the presence of γδ subsets in bronchoalveolar fluid supports the concept that peripheral depletion results at least in part from their recruitment to lung tissue during acute infection.

The single-cell transcriptomic approach has also been used to characterise tumour-infiltrating γδ T cells in virus-associated cancers, namely, HPV-associated head and neck squamous cell carcinoma and EBV-positive Hodgkin’s lymphoma, revealing an influence of the tumour’s viral status on infiltrating γδ cell characteristics [2].

The Special Issue also includes two reviews that focus on persistent infections with herpesviruses (HHVs), which strongly affect γδ T cell subsets. One focuses on viral infections following allogeneic stem cell transplantation used for the treatment of haematological malignancies in particular, where reactivations of CMV and EBV are common [3]. In this review, the interaction between conventional αβ T cells and γδ T cells for haematopoietic reconstitution, and the role of γδ T cells for the control of CMV/EBV reactivation as well as for the control of tumour relapse are discussed. In another paper [4], the literature was reviewed to question the role of carriage of different HHVs on γδ T cell responses occurring during acute or chronic co-infections with other pathogens, in particular, HIV or hepatitis viruses. Indeed, since HHVs are usually acquired early in life, persist throughout life in the host and can reactivate under multiple inflammatory conditions, their role is difficult to separate from that of co-infecting pathogens.

This Special Issue covers the current understanding of γδ T cells in viral infections. It is now clear that γδ T cells are in fact made up of several functionally different subsets that are involved in various ways in different viral contexts. Much remains to be done before we know precisely what this heterogeneous T cell population actually does and how the potential of individual subsets can be effectively exploited for immunotherapeutic purposes. Future work should clarify the beneficial and detrimental effects of their activation, which will most likely be possible due to the rapidly improving knowledge of their molecular targets and activation patterns. We hope that these contributions will help motivate a more systematic examination of these still puzzling cells in the assessment of immune status in viral infection. Finally, we would like to thank the authors who contributed to this Special Issue and the reviewers for their fruitful input.

## Data Availability

Not applicable.
